# Evaluating the Biomechanical Integrity of Various Constructs Utilized for First Metatarsophalangeal Joint Arthrodesis: A Systematic Review

**DOI:** 10.3390/ma16196562

**Published:** 2023-10-05

**Authors:** Abhinav R. Balu, Anthony N. Baumann, Terence Tsang, Grayson M. Talaski, Albert T. Anastasio, Kempland C. Walley, Samuel B. Adams

**Affiliations:** 1 Feinberg School of Medicine, Northwestern University, Chicago, IL 60208, USA; 2 College of Medicine, Northeast Ohio Medical University, Rootstown, OH 44272, USA; abaumann@neomed.edu; 3 Campbell University School of Osteopathic Medicine, Lillington, NC 27546, USA; t_tsang0821@email.campbell.edu; 4 Department of Orthopedics and Rehabilitation, University of Iowa, Iowa City, IA 52242, USA; grayson-talaski@uiowa.edu; 5 Department of Orthopaedic Surgery, Duke University, Durham, NC 27708, USA; albert.anastasio@duke.edu (A.T.A.); samuel.adams@duke.edu (S.B.A.); 6 Department of Orthopaedic Surgery, University of Michigan, Ann Arbor, MI 48109, USA; kcwalley@med.umich.edu

**Keywords:** MTP arthrodesis, biomechanics, hallux valgus, dorsal plate, interfragmentary screws

## Abstract

The first metatarsophalangeal (MTP) joint is a frequently loaded joint, handling loads up to 90% of bodyweight. First MTP arthrodesis is a frequently performed procedure designed to improve pain in patients with degenerative MTP joint disease. There are a wide variety of fixation constructs for this procedure without consensus on the most effective method. The purpose of this study was to compare the biomechanical integrity of various constructs utilized for first MTP arthrodesis. A systematic review of the literature was conducted in accordance with Preferred Reporting Items for Systematic Reviews and Meta-Analyses (PRISMA) guidelines. PubMed, CINAHL, MEDLINE, and Web of Science databases were searched from inception to 18 June 2023. Articles discussing the biomechanics of first MTP arthrodesis constructs were included. A total of 168 articles were retrieved. A total of 20 articles involving 446 cadaveric and synthetic bone constructs were included in the final review. Of the six articles comparing dorsal plating with compression screws to crossed interfragmentary screws, five found that dorsal plating had significantly higher stiffness. All three studies assessing shape-memory staples found them to be significantly less stable than crossed screws or dorsal plates alone. Both studies evaluating fully threaded screws found them to be stronger than crossed cancellous screws. Wedge resections have been shown to be 10 times stronger than standard planar or conical excision. Dorsal plating with compression screws is the gold standard for MTP arthrodesis. However, more research into newer methods such as fully threaded screws and wedge resections with an increased focus on translation to clinical outcomes is needed.

## 1. Introduction

The first metatarsophalangeal (MTP) joint is a frequently loaded joint that can be stressed up to 90% of body weight during each step [1,2,3,4,5]. First MTP arthrodesis is a commonly performed procedure intended to relieve pain and restore functionality to the foot for end-stage hallux rigidus and hallux valgus [6,7,8], the two most common pathologies of the first MTP joint [9,10]. Despite being a common procedure, union rates vary from 56% to 100% with hardware removal occurring in 8.5% of cases, indicating that further research is needed to improve overall patient outcomes [11,12,13]. A variety of fixation methods have been proposed to increase rates of union and joint stabilization such as interfragmentary and intramedullary screws, dorsal and medial plates, crossed K-wires, staples, external fixation devices, wire sutures, and a variety of combinations of the aforementioned constructs [14,15,16,17,18]. In addition, there has been increased interest in identifying the optimal form of joint resection prior to arthrodesis to maximize joint surface area and stability [19,20,21].

Despite the growing interest within the literature and the variety of fixation methods, there currently exists no consensus on the ideal fixation method for first MTP arthrodesis or the biomechanical underpinnings of these treatments [1,9,19,22,23,24,25]. In the past decade alone, there have been 12 published primary studies describing the biomechanics of the first MTP joint [1,11,13,19,22,23,25,26,27,28,29,30], warranting a systematic analysis of the existing literature. However, to date, no systematic review has synthesized and evaluated the biomechanical properties of the variety of fixation and joint resection methods described in the literature for first MTP arthrodesis. As such, the purpose of this systematic review is to evaluate the biomechanical properties of the fixation constructs described in the literature to better elucidate which constructs offer the greatest joint stability and, thus, may be best suited to handle postoperative weightbearing and maximize functionality to ultimately improve patient outcomes.

## 2. Materials and Methods

### 2.1. Initial Search Set-Up

This systematic review was designed in accordance with Preferred Reporting Items for Systematic Reviews and Meta-Analyses (PRISMA) guidelines. A full search of PubMed, CINAHL, MEDLINE, and Web of Science databases was conducted from inception to 18 June 2023. Search terms used in each database were (MTP OR MTPJ OR metatarsophalangeal OR metatarsal-phalangeal OR hallux valgus OR hallux rigidus) AND biomechanic* AND (fusion OR arthrodesis). Duplicates were then removed prior to title and abstract screening for relevance. Approved full texts were then reviewed in their entirety for compliance with inclusion/exclusion criteria. Data extraction was then performed on the included full-text articles.

### 2.2. Inclusion/Exclusion Criteria

Inclusion criteria were articles with evidence level I–III and studies concerning arthrodesis of the first MTP joint and reporting on biomechanical properties of the fixation construct. Exclusion criteria were abstract only, full-text not available, non-English articles, case reports, case series, technical notes, systematic reviews, and meta-analyses and book chapters.

### 2.3. Study Definitions

Strength and failure load are interchangeable terms used to characterize the maximum load that a given structure can withstand before undergoing irreversible deformation or failure. Stiffness, on the other hand, pertains to the inherent elastic characteristics of a structure. This value refers to a quantification of the resistance to deformation under an applied force, indicating the amount of force per unit length that can be applied without causing permanent distortion.

### 2.4. Data Extraction

Data extracted from articles selected for this systematic review included first author of article, full title of article, year of article publication, construct type (cadaver, synthetic bone analogue, 3D printed bone materials), number of constructs used, fixation method (plate, screw, K-wire, staples), and functional outcome measures related to the MTP arthrodesis (plate stiffness, load to failure, mode of failure, plantar gap, cycles completed, etc.).

### 2.5. Statistical Analysis

Statistical analysis was performed in Microsoft Excel version 16.77.1. This systematic review was performed using a narrative approach as study heterogeneity prevented meta-analysis. Therefore, only descriptive statistics and basic frequencies were calculated.

## 3. Results

### 3.1. Initial Search Results

**Figure 1:**
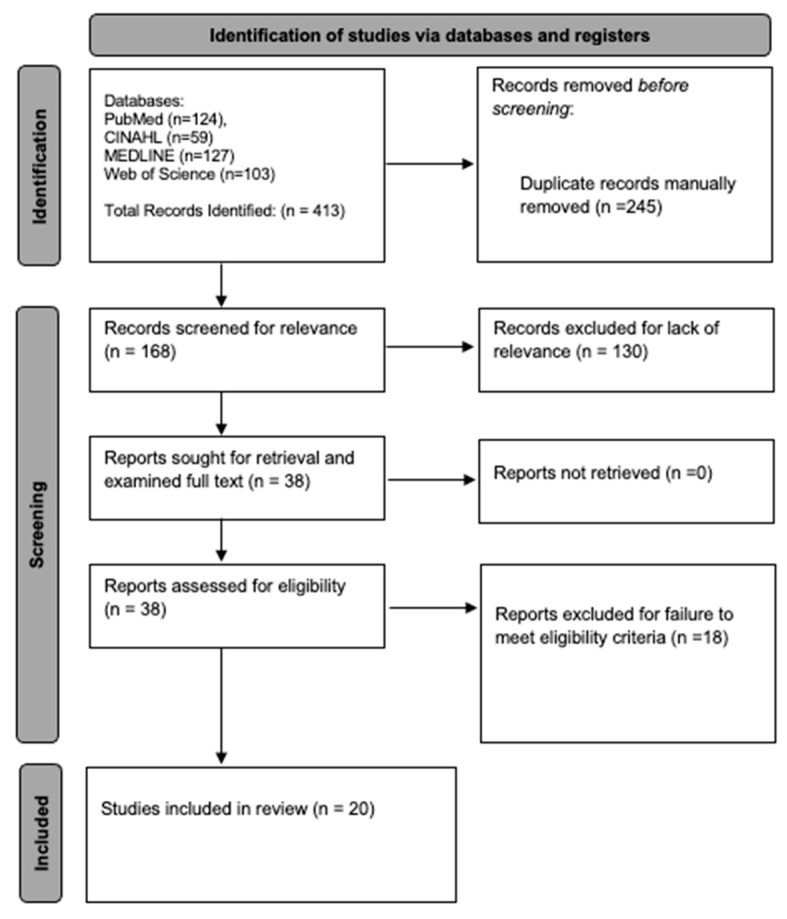
PRISMA diagram for this systematic review of the biomechanics of first MTP joint arthrodesis. Databases searched, article screening with reason for exclusion, and final article count are displayed.

The initial search yielded 413 articles, 245 of which were duplicates, resulting in a total of 168 unique articles. Of these, 130 were excluded for a lack of relevance, leaving 38 articles for full text review. A further 18 articles did not meet the eligibility criteria following full-text review and were thus excluded. Figure 1 below outlines the search process and article identification for the current systematic review.

### 3.2. Study Demographics

Across the 20 included articles, there were a total of 143 pairs of human cadaveric toes, 16 pairs of porcine cadaveric toes, and 92 synthetic bone models for a total of 446 cases of first MTP arthrodesis [1,9,11,13,19,22,23,24,25,26,27,28,29,30,31,32,33,34,35,36]. Arthrodesis was achieved using a variety of modalities including dorsal plates, interfragmentary lag screws, K-wires, wire sutures, and staples. Four articles utilized exclusively plate fixation while two studies utilized only screw-based constructs. Only one study utilized shape-memory staples alone. The remaining twelve studies compared a mix of the aforementioned constructs. One article did not describe the mode of fusion. Table 1 below shows an itemized breakdown of each included article.

### 3.3. Plate Fixation:

Four studies exclusively assessed the effectiveness of various plating techniques in first MTP arthrodesis [1,11,13,27]. Mandell et al. compared the “gold standard”, a dorsal non-locking plate with lag screw, to a locking compression plate with and without a lag screw and found that both locking compression plates had significantly higher stiffness (*p* < 0.05), and locking compression plates without lag screw had significantly less displacement when compared to the gold standard (3.91 mm vs. 7.91 mm, *p* = 0.015) [1]. Hunt and colleagues corroborated these findings, demonstrating that locked plates experience significantly less plantar gapping than non-locked plates during fatigue endurance testing from cycles 10,000 to 250,000 of a 90 N, 250,000 cycle load period designed to simulate 6 weeks of postoperative weightbearing [11]. In this study, the locked plates also demonstrated significantly higher stiffness. There were no differences in arthrodesis failure rates or load to failure between the two groups. Witkowski and colleagues evaluated the relatively novel concept of medial plating by comparing it to traditional dorsal plating. They found that medial plating provides more stiffness and resistance to dorsal displacement (9.63 mm vs. 19.60 mm) and that medial plates experience less overall mechanical stress [13]. Aiyer et al. studied the application of a dorsal dual-miniplate compared to a standard dorsal plate but found no significant difference in stiffness or strength [27].

### 3.4. Screw Fixation

Molloy et al. explored the strength of intramedullary screws for first MTP arthrodesis relative to standard techniques using crossed interfragmentary screws, finding that intramedullary screw fixation was significantly stiffer than interfragmentary screw fixation (18.7 ± 10.1 vs. 10.2 ± 6.1 N/mm) [37]. Intramedullary screws were also 50% stronger when measured using load to failure than interfragmentary screws (149.2 ± 88.2 vs. 100.2 ± 70.8 N), although this difference was not statistically significant. Rongstad et al. similarly demonstrated that cannulated intramedullary screws were significantly stronger (failure load) than AO oblique cancellous screws (154 ± 88.3 vs, 66.7 ± 18.5N, *p* = 0.03), but did not demonstrate any significant difference between the AO screws and Steinmann pins [33]. Lucas and colleagues studied the properties of the screws themselves, comparing triple-threaded headless screws to partially threaded lag screws [25]. Triple-threaded screws required a significantly higher failure load compared to partially threaded lag screws (124.94 ± 53.33 N vs. 96.45 ± 54.82 N, *p* = 0.040) and achieved significantly higher bending stiffness (13.13 ± 6.15 N/mm vs. 8.51 ± 5.03 N/mm, *p* = 0.017).

Sykes and Hughes studied the strength of AO cancellous screw fixation relative to horizontal and vertical wire sutures as well as an external fixation device [34]. The AO cancellous screw demonstrated the highest stiffness (23.7 N/mm) followed by external fixator (5.0 N/mm), vertical wire (2.3 N/mm), and horizontal wire (2.2 N/mm). In a slightly different experimental design, Faraj et al. compared screw fixation to circumferential wire fixation [35]. Screw fixation was six times as strong as circumferential wire fixation; however, both demonstrated equal elasticity over similar displacement ranges.

### 3.5. Staple Fixation

Wilmott et al. studied shape-memory staples by comparing a single vertical or horizontal staple, orthogonal paired 0–90° staples, paired 45–135° staples, and crossed screws. All staple configurations other than the paired 45–135° staples failed at significantly lower loads than the crossed screws (*p* < 0.001). There was no significant difference between the paired 45–135° staples and the crossed screws; however, no construct was strong enough for immediate weight bearing. Neufeld et al. also compared shape-memory staples to cross-cannulated screws as well as a dorsal plate, finding that the staples did indeed fail at significantly lower loads than the crossed-cannulated screws (*p* < 0.029) and the dorsal plate (*p* < 0.002) [36]. Schafer et al. similarly compared orthogonal nitinol staples to a dorsal staple with a crossed screw [30]. All eight specimens implanted with orthogonal staples failed at an average of 37 ± 81 cycles of a 90 N, 250,000 cycle protocol designed to simulate 6 weeks of postoperative weightbearing due to excessive gap formation > 7 mm. In total, 7/8 specimens fixed with a dorsal staple and crossed screw failed at an average at 14,900 ± 39,000 cycles. The number of cycles to failure was significantly lower in the orthogonal staple group (*p* < 0.05) with both groups demonstrating biomechanical instability consistent with clinical failure.

### 3.6. Screw vs. Plate Fixation:

Seven studies directly compared plate-to-screw fixation for first MTP arthrodesis (Table 2).

Campbell et al. found that a dorsal plate with lag screw was significantly stiffer than crossed lag screws or plating alone in synthetic bone models (*p* < 0.008) [23]. However, when comparing a dorsal plate with lag screw to crossed screws in a cadaver model, there was no significant difference in stiffness (*p* = 0.08) or load to failure (*p* = 0.296), with the crossed screw construct exhibiting the higher overall stiffness but a lower overall failure load. Buranosky and colleagues further evaluated the dorsal plate with lag screw to the crossed screw construct in a cadaveric model [31]. The dorsal plate with lag screw was significantly stiffer (121 N/mm vs. 72 N/mm, *p* < 0.01) during the first millimeter of displacement between the metatarsal head and the proximal phalanx and required a significantly higher failure load than the crossed screws (180 N vs. 130 N, *p* < 0.002).

Similarly, Foote and colleagues found that dorsal plating with compression screws was significantly stiffer than compression screws or locking plate alone (*p* = 0.032) and was also able to withstand significantly more cycles of loading (*p* < 0.001) intended to mimic postoperative weightbearing [26]. Consistent with these results, Harris et al. found dorsal plating with interfragmentary lag screws to have significantly higher stiffness than crossed screws or dorsal plates alone (*p* < 0.001) but no significant difference in failure load (*p* = 0.93) [28]. Fuld et al. conducted a slightly different analysis evaluating arthroscopic insertion of fully threaded screws compared to dorsal plating with compression screws [22]. The fully threaded screws demonstrated significantly greater stiffness (51.7 N/mm vs. 31.6 N/mm, *p* = 0.0045), but no significant difference in plantar gapping or load to failure. Politi et al. found that the lag screw and dorsal plate was, by a large margin, the strongest construct, requiring the largest applied moment arm to open the joint space (4958.5 N-Mm) [24]. This value was almost three times larger than the lag screw with conical excision and 10 times larger than the K-wire or dorsal plate alone.

### 3.7. Type of Osteotomy

Fifteen of the twenty included studies performed joint osteotomy as part of the simulated arthrodesis procedure. Three studies performed a flat or planar excision, seven studies performing conical reaming or the creation of a hemispherical surface for a cup–cone fit, and the remaining five studies compared various methods of joint fixation. Politi et al. compared a wide variety of fixation methods including interfragmentary lag screw alone and with conical and planar surface excision, crossed K-wires, dorsal plate alone, and lag screw plus dorsal plate [24]. Planar excision produced a significantly stronger arthrodesis when compared to conical excision. Sykes and Hughes had similar results when comparing planar excision of the MTP joint to dome surfacing. Planar MTPJ with cancellous screw had a significantly higher failure load than dome shaped MTPJ with cancellous screw (*p* = 0.03) [34]. These results are in contrast to Curtis and colleagues’ work demonstrating that conical reaming followed by interfragmentary screw placement was significantly stronger (*p* < 0.03) and more stable (*p* < 0.05) than dorsal plating or interfragmentary screws alone following planar excision [32]. Unlike the prior studies, Harris et al. found no significant difference between planar and conical osteotomy (*p* = 0.99) [28].

Barták et al. studied the wedge, planar, and ball-and-socket surface excision methods, assessing each via the reactive force necessary for separation, surface contact area, and final length of the first ray [19]. They found that ball-and socket-preparations required the least reactive force to separate and had the least surface area, while flat resection demonstrated the greatest surface area. Both 90° and 100° wedge resections demonstrated the least shortening of the first ray and required greater than 10 times the reactive force needed for the planar and ball-and-socket excisions, indicating that these constructs may be more stable and sustainable.

## 4. Discussion

First MTP arthrodesis is a commonly utilized procedure for end-stage hallux rigidis and hallux valgus with non-union rates reported as high as 30% [1]. A variety of fixation methods have been implemented in an effort to increase joint stability and functional capacity following arthrodesis. However, there remains a lack of consensus regarding the most effective method of fixation. This systematic review is the first and only of its kind to synthesize all existing biomechanical data regarding the various methods of fixation to assess which constructs maximize biomechanical stability and load to failure. Across the 20 articles reviewed, there were four main forms of fixation identified: dorsal plating, crossed interfragmentary screws, shape-memory staples, and dorsal plating with compression screws. Our review demonstrates that dorsal plating with compression screws is the most stable fixation construct. When considering screw fixation alone, intramedullary and fully threaded screws may offer superior strength and stability compared to cancellous interfragmentary screws. Finally, the method of joint preparation is an important determinant of arthrodesis stability and an area in need of more research.

Mandell and colleagues described dorsal plates with compression screws as the gold standard [1]. Our study largely confirms this statement with five of the six studies directly comparing dorsal plating with compression screws to crossed screw fixation, finding that dorsal plating with compression screws had significantly higher stiffness [23]. In three of the studies, the failure load was also significantly higher for the dorsal plate with compression screw construct [24,26,31]. One study reported higher stiffness for a simulated arthroscopic insertion technique for fully threaded crossed screws [22]. However, there was no significant difference in load to failure or plantar gapping. Of note, Politi et al. found dorsal plating alone to be ineffective in resisting excessive dorsiflexion of the first MTP joint, a commonly occurring complication as reported by DeOrio [24,38]. Interfragmentary screw placement, therefore, may augment the stability provided by a dorsal plate, and produce a more resistant fixation. Clinical analysis of dorsal plating without interfragmentary screws demonstrates similar results, with multiple studies finding no difference in fusion rates between dorsal plating alone and crossed screw fixation [17,39,40]. When evaluating the type of screws used to provide intramedullary compression, fully threaded screws are significantly stronger and stiffer than standard screws [25]. While simulated arthroscopic insertion of fully threaded screws demonstrated comparable stiffness to dorsal plating with standard lag screws in a single study, a more direct comparison between crossed fully threaded compression screws and a dorsal plate with fully threaded compression screw is needed to properly evaluate the two methods.

In addition to identifying the most biomechanically sound fixation constructs, our study also sought to evaluate newer fixation constructs and novel applications of existing constructs. Shape-memory staples are a more recently introduced fixation construct manufactured from metal alloys that allow for continuous compression when heated to body temperature, referred to as dynamic compression [29]. Our study shows that shape-memory staples are significantly less biomechanically sound than crossed screws or dorsal plates. All three studies utilizing shape-memory staples found that the staples tolerated significantly less load prior to failure when compared with crossed screws, dorsal plate, or a dorsal plate with compression screw, indicating that these devices may not be equipped to sustain early postoperative weightbearing. Another newer construct design was the use of a single intramedullary screw in place of oblique interfragmentary screws. Both studies utilizing a single intramedullary screw found it to be either significantly stiffer or significantly stronger than the interfragmentary screws [33,37]. Single intramedullary screws cross perpendicular to the joint axis and are able to provide support along their axis, whereas crossed screws enter the arthrodesis site at an angle, providing less stability at points of contact. While more data is needed, our review of existing data indicates that intramedullary screws are the most stable option when considering screw fixation alone. As mentioned, intramedullary single screw fixation using fully threaded compression screws may provide even greater stability and should be evaluated in further comparative studies.

A key factor in determining the success of the arthrodesis is the method of joint preparation. Of the twenty included studies, five compared various methods of joint preparation prior to arthrodesis. There are three main methods of joint preparation: simple cartilage excision, planar or flat-on-flat excision using an oscillating saw, or ball-and-socket excision using conical reamers intended to create congruent surfaces [19]. Two studies found planar excision to be significantly stronger than conical reaming [24,34] whereas Curtis et al. found conical reaming to be significantly stronger [32] and Harris et al. found no significant difference (*p* = 0.99) [28]. However, in a subsequent group analysis, Curtis et al. found no difference between conical reaming and planar excision with K-wire fixation. It is worth noting that Harris et al. used dorsal plating with lag screws for fixation while the other studies fixed with interfragmentary screws. Bartak and colleagues were the only group to report on wedge resections, finding that wedge preparations of the joint were more than 10 times as strong as planar resections, which were more stable than ball-and-socket resections [19]. Planar resection provided the greatest joint surface area. Thus, while the limited existing data indicate that planar excision leads to a more stable joint, especially when followed by interfragmentary screw fixation, more research on the promising new technique of wedge resections and directly comparing wedge and planar resections across a variety of fixation constructs is needed.

Clinical studies evaluating MTP arthrodesis success validate the biomechanical findings of this systematic review [17,41,42]. In a study of 46 patients undergoing MTP arthrodesis with a dorsal plate and intramedullary screw, Kumar and colleagues found that 98% achieved successful fusion with 100% patient satisfaction and an average time to fusion of 3 months [41]. Likewise, in a study of 49 patients that underwent MTP arthrodesis with a dorsal plate and compression screw, Goucher and Coughlin reported a 92% fusion rate and a 96% satisfaction rate with significant improvement in AOFAS and pain scores following the arthrodesis procedure [21]. In a larger cohort of 60 patients, Chraim et al. reported dorsal plating with compression screws to be the modality of choice for MTP arthrodesis with fusion rates approaching 95% as well as significant improvements in AOFAS scores [42]. In each of the aforementioned studies, osteotomy was performed using conical reamers to produce a cup–cone joint surface interface. While biomechanical data demonstrate a greater joint surface area following planar resection, conical reaming may allow for more flexibility when aligning the joint surface in three-dimensional space and therefore provide greater precision when placing fixation constructs about the joint. Unlike the aforementioned studies, Bennett and colleagues reported an 87% fusion rate and 13% hardware failure rate in a study of 95 patients undergoing arthrodesis with a dorsal plate and intramedullary screw [43]. The high rate of hardware complication and poor outcomes are likely due to the usage of dorsal plates designed for use in hand procedures. Plates designed to maintain the anatomical contours of the foot and preserve natural kinematics are necessary to maximize functional outcomes postoperatively [38]. Existing clinical data demonstrate that conical reaming followed by dorsal plating with compression screws provides a high rate of fusion when performed with foot-specific plates. More data in the form of randomized control trials comparing dorsal plating with compression screws to other fixation modalities are needed to validate efficacy, however.

This systematic review has several limitations that impact its conclusions and subsequent implications. The primary limitation of this study was the large variety in experimental design and lack of clinical validity. Arthrodesis was performed on a multitude of different models and materials, resulting in varied measurements for identical constructs across the various models. Furthermore, the majority of included articles had small sample sizes with limited discussion of clinical applicability. Included articles assessed first MTP biomechanics without considering how patient comorbidities or behaviors would impact construct stability, as evidenced by only two studies utilizing a cyclic loading protocol intended to simulate postoperative weightbearing. Second, outcome parameters and measurement techniques were not standardized across included articles. For example, some studies chose to report on stiffness while others chose to assess failure load or plantar gapping. This prevented meta-analysis due to study heterogeneity as there was not adequate overlap between primary outcome parameters. Amongst those studies that did share outcome parameters, such as stiffness, some defined and measured stiffness as the initial slope of the force–displacement curve whereas others reported on bending stiffness or elastic stiffness. This heterogeneity limits our ability to perform higher-level numerical analysis of these data points. Third, it must be noted that these findings are solely in the context of biomechanical analysis. Functional movement does not strictly respect planar dimensions and is often difficult to replicate accurately. Furthermore, factors such as operative feasibility and cost are important considerations that cannot be ignored when making a clinical decision regarding construct choice. Finally, certain fixation/joint preparation methods such as fully threaded screw, medial plates, and wedge resections were underrepresented in our systematic review. While this may be because these fixation methods are less commonly used in clinical practice, more research is needed to evaluate the process of first MTP arthrodesis, from joint preparation to construct insertion, to determine which procedure is best suited to maximize postoperative outcomes.

## 5. Conclusions

Dorsal plating with compression screws is the most biomechanically stable fixation construct amongst existing methods for MTP arthrodesis. Fully threaded and intramedullary screws are newer methods with promising results that warrant further study. Joint excision methods are imperative in determining arthrodesis success and must be further studied as well. Overall, higher-level research in the form of randomized control trials with translation to clinical outcomes is needed to acquire a more practical understanding of these fixation methods for MTP arthrodesis.

## Figures and Tables

**Table 1:** Information about each study included in this systematic review. Author, full title, year of publication, model type used to perform the arthrodesis, number of models used, and primary fixation method are shown.

First Author (Year)	Model (Cadaver, Computer, etc.)	Number of Constructs (n)	Primary Fixation Method
Schafer (2022) [30]	Cadaver	8	Shape Memory Staples
Foote (2012) [26]	Synthetic Bone	24	Multiple
Aiyer (2015) [27]	Cadaver	24	Dorsal Plate
Curtis (1993) [32]	Cadaver	20	Multiple
Molloy (2003) [37]	Cadaver	10	Screw
Rongstad (1994) [33]	Cadaver	36	Multiple
Buranosky (2001) [31]	Cadaver	24	Multiple
Politi (2003) [24]	Synthetic Bone	40	Multiple
Hunt (2012) [11]	Cadaver	18	Locking Plate
Campbell (2017) [23]	Cadaver + Synthetic Bone	5	Multiple
Lucas (2014) [25]	Cadaver	22	Screw
Harris (2017) [28]	Synthetic Bone	20	Multiple
Willmott (2018) [29]	Porcine Cadaver	32	Multiple
Witkowski (2021) [13]	Synthetic Bone	2	Plate
Faraj (2007) [35]	Synthetic Bone	2	Multiple
Bartak (2020) [19]	Synthetic Bone	4	N/A
Fuld (2019) [22]	Cadaver	11	Multiple
Mandell (2018) [1]	Cadaver	24	Plate
Neufeld (2002) [36]	Cadaver	42	Multiple
Sykes (1986) [34]	Cadaver	15	Multiple

**Table 2:** Construct stiffness and load to failure data from the studies that directly compared plate and screw fixation with respective *p*-values included.

First Author (Year)	Stiffness	Load to Failure
Curtis (1993) [32]	Group 1: Conical Excision Interfragmentary Screw: 0.69 ± 0.32 N/mm (0.24–1.0) * Dorsal Plate: 0.17 ± 0.08 N/mm (0.05–0.26) * Planar Excision Interfragmentary Screw: 0.28 ± 0.11 N/mm (0.15–0.42) * Group 2: Conical Excision Interfragmentary Screw: 0.28 ± 0.18 N/mm (0.12–0.58) K Wire: 0.28 ± 0.16 N/mm (0.08–0.50) * = *p* < 0.03	Group 1: Conical Excision Interfragmentary Screw: 91.2 ± 28.2 (575–23) * Dorsal Plate: 20.6 ± 4.6 (12.4–24) * Planar Excision Interfragmentary Screw: 28.2 ± 3.3 (24.2–31) * Group 2: Conical Excision Interfragmentary Screw: 79.4 ± 25.9 (41.3–110) * K Wire: 40.2 ± 14.3 (19–57.1) * * = *p* < 0.01, All measurements N^−1^
Buranosky (2001) [31]	MT1 Displacement (0–1 mm): Dorsal Plate/Screws: 121 ± 56 N/mm * Crossed Screws = 72 ± 33 N/mm * MT1 Displacement (1–2 mm): Dorsal Plate/Screws: 37 ± 10 N/mm Crossed Screws: 31 ± 16 N/mm * = *p* < 0.01	Dorsal Plate Lag Screw: 180 ± 58 * Crossed Screw: 130 ± 64 * * = *p* < 0.002
Politi (2003) [24]	N/A	Mean Moment (N-mm): Dorsal Plate Lag Screw: 4958.5 * Planar Excision Interfragmentary Screw: 3030.2 * Conical Excision Interfragmentary Screw: 1870.2 * Dorsal Plate: 397.5 K wire: 304.5 * = All constructs significantly differ from each other except for Dorsal Plate and K-wire
Campbell (2017) [23]	Synthetic Bone Model: Non-Locking Plate: 15.6 ± 1.7 N/mm * Locking Plate: 18.1 ± 2.3 N/mm * Dorsal Plate Lag Screw: 373.4 ± 76.3 N/mm * Crossed Screws: 94.7 ± 12.5 N/mm * Cadaveric Model: Dorsal Plate Lag Screw = 122.1 ± 5.9 N/mm Crossed Screws = 152.4 ± 14.2 N/mm * = All constructs significantly differ from each other (*p*< 0.008) except for Non-Locking and Locking Plates	Synthetic Bone Model: Dorsal Plate Lag Screw: 130.9 ± 19.4 N Crossed Screws: 101 ± 17.8 N Cadaveric Model: Dorsal Plate Lag Screw: 154.1 ± 40.7 N Crossed Screws: 93.9 ± 14.4 N
Harris (2017) [28]	Conical Excision Dorsal Plate Lag Screw: 19.30 ± 1.43 N/mm Planar Excision Dorsal Plate Lag Screw: 19.08 ± 2.89 N/mm Crossed Screws: 9.32 ± 2.17 N/mm * Dorsal Plate: 3.76 ± 0.55 N/mm * * = significantly weaker than both dorsal plating methods *p* < 0.001	Conical Excision Dorsal Plate Lag Screw: 364.4 ± 25.9 N Planar Excision Dorsal Plate Lag Screw: 360.6 ± 31.5 N Crossed Screws: 382.4 ± 84.6 N Dorsal Plate: 211.5 ± 21.4 N * * = *p* < 0.001, significantly lower than all other groups
Fuld (2019) [22]	Dorsal Plate Lag Screw: 31.6 ± 25.09 N/mm * Fully Threaded Compression Screw: 51.7 ± 27.21 N/mm * * *p* = 0.0045	Dorsal Plate Lag Screw: 198.6 ± 33.68 N Fully Threaded Compression Screw: 290.31± 95.69 N

## Data Availability

Not applicable.
